# Identification of thioredoxin-1 as a biomarker of lung cancer and evaluation of its prognostic value based on bioinformatics analysis

**DOI:** 10.3389/fonc.2023.1080237

**Published:** 2023-01-27

**Authors:** Xiaoting Liu, Xilin Dong, Yaxin Hu, Yanan Fang

**Affiliations:** Department of Respiratory, The Second Affiliated Hospital of Xi’an Jiaotong University, Xi’an, China

**Keywords:** thioredoxin-1, lung cancer, oxidative stress, bioinformatics, survival

## Abstract

**Background:**

Thioredoxin-1 (TXN), a redox balance factor, plays an essential role in oxidative stress and has been shown to act as a potential contributor to various cancers. This study evaluated the role of TXN in lung cancer by bioinformatics analyses.

**Materials and methods:**

Genes differentially expressed in lung cancer and oxidative stress related genes were obtained from The Cancer Genome Atlas, Gene Expression Omnibus and GeneCards databases. Following identification of TXN as an optimal differentially expressed gene by bioinformatics, the prognostic value of TXN in lung cancer was evaluated by univariate/multivariate Cox regression and Kaplan–Meier survival analyses, with validation by receiver operation characteristic curve analysis. The association between TXN expression and lung cancer was verified by immunohistochemical analysis of the Human Protein Atlas database, as well as by western blotting and qPCR. Cell proliferation was determined by cell counting kit-8 after changing TXN expression using lentiviral transfection.

**Results:**

Twenty differentially expressed oxidative stress genes were identified. Differential expression analysis identified five genes (*CASP3, CAT, TXN, GSR*, and *HSPA4*) and Kaplan–Meier survival analysis identified four genes (*IL-6, CYCS, TXN*, and *BCL2*) that differed significantly in lung cancer and normal lung tissue, indicating that TXN was an optimal differentially expressed gene. Multivariate Cox regression analysis showed that T stage (T3/T4), N stage (N2/N3), curative effect (progressive diseases) and high TXN expression were associated with poor survival, although high TXN expression was poorly predictive of overall survival. TXN was highly expressed in lung cancer tissues and cells. Knockdown of TXN suppressed cell proliferation, while overexpression of TXN enhanced cell proliferation.

**Conclusion:**

High expression of TXN plays an important role in lung cancer development and prognosis. Because it is a prospective prognostic factor, targeting TXN may have clinical benefits in the treatment of lung cancer.

## Introduction

Lung cancer is one of the most prevalent cancers worldwide. The National Cancer Database and the American Cancer Society have estimated that, in 2022, lung cancer was the tenth most prevalent cancer in men and the seventh most prevalent in women (1), while also being the leading cause of cancer deaths in both (2). Recent advances in molecular targeted therapies and immunotherapeutic agents have prolonged survival in lung cancer patients (3, 4), although the effects of these treatments differ among histological subtypes. Agents targeting driver genes have been reported to contribute substantially to reductions in lung cancer mortality (5). Identifying new potential driver genes can provide support for the diagnosis and management of lung cancer.

Analyses of big data through gene sequencing, chip technology and bioinformatics have been utilized for the identification of disease characteristics and the rapid development of medicines to treat these diseases. Databases containing information on large numbers of patients have shown promise in analyzing the characteristics of various diseases, especially cancers (6). Analyses of databases such as The Cancer Genome Atlas (TCGA), Gene Expression Omnibus (GEO), and Human Protein Atlas (HPA), which contain data on patients with many types of cancer, have made possible the identification of biomarkers for the more effective diagnosis and management of cancer patients.

Thioredoxin-1 (Trx1, TXN), a member of the thioredoxin family, is a regulator of redox balance through the dynamic transformation between its oxidized and reduced states (7). TXN is highly expressed in various cancers and regulates cell proliferation and apoptosis through different signaling pathways (8). Small molecule inhibitors of the thioredoxin system have shown significant tumor-suppressing effects, suggesting that TXN may be a potential therapeutic target in cancer treatment (9).

The specific mechanisms of action of TXN in lung cancer have not been determined. The present study utilized bioinformatics methods to identify key genes related to lung cancer and oxidative stress. These analyses showed that TXN was differentially expressed in lung cancer and normal lung tissue and that TXN had prognostic characteristics in lung cancer. The associations of TXN expression with patient survival and prognosis were analyzed, and its prognostic value was verified *in vitro*.

## Materials and methods

### Data acquisition

**Figure 1:**
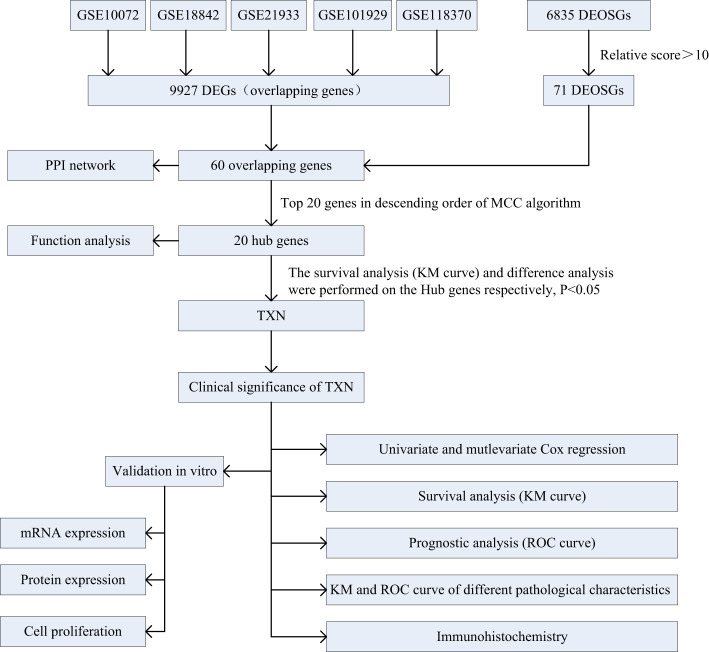
Flowchart of the study design.

Five lung cancer related datasets, GSE10072, GSE18842, GSE21933, GSE101929, and GSE118370, were selected from GEO database (https://www.ncbi.nlm.nih.gov/geo), and the gene expression profiles and clinical characteristics of each dataset were downloaded. Data characteristics were assessed using the GEO2R tool, which generated uniform manifold approximation and projection (UMAP) and volcano diagrams. Lung cancer RNAseq data were obtained from the TCGA database (https://portal.gdc.cancer.gov/), and genes related to the oxidative stress phenotype in lung cancer were obtained from the GeneCards database (https://www.genecards.org/). Information on immunohistochemical determination of TXN expression of lung cancer and normal lung epithelial tissues were obtained from HPA database (https://www.proteinatlas.org/). The specific analytic process is shown in **Figure 1**.

### Identification of differentially expressed oxidative stress genes

Analysis of the five GSE datasets identified 9927 overlapping genes, which were defined as differentially expressed genes (DEGs). A total of 9629 oxidative stress genes (OSGs) were downloaded from the GeneCards database. Only 71 OSGs were selected based on the relative scores > 10. Further analysis of the DGEs and OSGs together identified 60 differentially expressed oxidative stress genes (DEOSGs).

### Protein-protein interaction (PPI) network and hub genes

Interacting nodes of the 60 DEOSGs were determined by uploading these 60 DEOSGs to the STRING database (https://cn.string-db.org/); the interaction score thresholds were adjusted to 0.7 to ensure high reliability of intermolecular interactions. The PPI network was visualized using the Cytoscape database. Cytohubba is a plug-in component in the Cytoscape database to screen hub genes. The screening criteria were defined as the top 20 genes in descending order on the maximum correlation coefficient (MCC) algorithm. Thus, these 20 genes were identified as hub genes.

### Biological function enrichment and pathway analysis

Gene Ontology (GO) and Kyoto Encyclopedia of Genes and Genomes (KEGG) enrichment analyses of hub genes were assessed using the Xiantao website (https://www.xiantao.love/), a tool for data analysis and visualization. The Gene Set Enrichment Analysis (GSEA) database (http://www.gsea-msigdb.org/gsea/) was used for analyses of the Hallmark, C2 and C5 gene sets in the TCGA dataset. The threshold for significant enrichment was defined as a false discovery rate (FDR) < 0.25 and a p.adjust < 0.05.

### Univariate/multivariate Cox regression and prognostic analysis

The 20 hub genes in the five GSE datasets were subjected to differential analysis of expression, whereas the associations of the 20 hub genes in the TCGA dataset with survival were analyzed by the Kaplan-Meier (KM) method. TXN expression was found to differ significantly in each of the GSE and TCGA datasets, identifying TXN as a target gene. The relationships between TXN expression and the clinicopathological characteristics and overall survival (OS) of patients in these datasets were analyzed by univariate and multivariate Cox regression analyses. The prognostic value of TXN expression was analyzed using a KM plotter and by receiver operating characteristic (ROC) curve analysis.

### Cell culture

The H23, A549, H1299 and PC9 human lung cancer cell lines were cultured in RPMI 1640, and the bronchial epithelial cell line BEAS-2B was cultured in DMEM, both supplemented with 10% fetal bovine serum (FBS; cat. no. 16140071, Gibco) and 1% penicillin-streptomycin (cat. no. 15140122, Gibco) at 37°C in a 5% CO_2_ incubator.

### Western blotting

Cells were lysed and total protein extracted with RIPA buffer (cat. no. 87787, Thermo Fisher Scientific) containing protease and phosphatase inhibitor. Protein concentrations were measured by the BCA method. Each sample was added 5% loading buffer (cat. no. BL529B, Biosharp), and the samples were incubated in boiling water for 5 min. Total proteins were separated on 12% SDS-PAGE gels (cat. no. PE001, Zhonghuihecai) and transferred to PVDF membranes (cat. no. IPFL00010, Millipore). The membranes were incubated in quick blocking liquid (cat. no. P0220, Beyotime) for 5-10 min at room temperature, followed by incubation with primary antibodies overnight at 4°C. The membranes were washed three times with TBST for 15 min each, incubated with secondary antibodies for 1 h at room temperature, and washed three times with TBST for 15 min each. Bands were detected using ECL chemiluminescent kits (cat. no. BL520A, Biosharp). The primary antibodies included rabbit anti-Trx-1 (diluted 1:1000, Cat. No. 14999-1-AP, RRID: AB_2272597), rabbit anti-GAPDH (diluted 1:100000; Cat. No. 60004-1-Ig, RRID: AB_2107436) and rabbit anti-actin (diluted 1:1000; Cat. No. 20536-1-AP, RRID: AB_10700003).

### Quantitative polymerase chain reaction (qPCR)

Total RNA was extracted from cells using TRIzol reagent (cat. no. RK30129, ABclonal), and cDNA was synthesized using ABScript III RT Master Mix Kits (cat. no. RK20428, ABclonal), following the manufacturer’s instructions. qPCR was performed using primers for TXN (forward, 5’-GTGAAGCAGATCGAGAGCAAG-3’; reverse, 5’-CGTGGCTGAGAAGTCAACTACTA-3’) and β-actin (forward, 5’-CCTTCCTGGGCATGGAGTC-3’; reverse, 5’-TGATCTTCATTGTGCTGGGTG-3’). The amplification conditions consisted of an initial denaturation at 95°C for 3 min, followed by 40 cycles of denaturation at 95°C for 5 s and annealing and extension at 60°C for 32 s.

### Lentiviral transfection

Lentiviral transfection experiments were performed following manufacturer instructions. First, cells were seeded at a density of 30% in 6-well plates; After 24 h, complete medium containing a defined amount of virus solution was added into per well, which was removed after 16-24 h transfection; Cells were incubated at 37°C in a 5% CO_2_ incubator for 48-72h. Then, a fluorescence microscope was used to observe the transfection effect; Finally, puromycin with 2 ug/ml was added into the complete medium in order to select the stable transfectant cell lines. RAN and protein of cells were extracted to verify the transfection effect.

### CCK8 assay

Cell proliferation ability was measured using Cell Counting Kit-8 (CCK8; cat. no. C0038, Beyotime). Lentivirus-transfected cells were seeded into 96-well plates and incubated for 24 h, 48h, 72h and 96h at 37°C in a 5% CO_2_ incubator. Later, 100 ul serum-free medium with 10% CCK-8 were added to per well. After 1-1.5 h at 37°C, the absorbance was detected at 450 nm.

### Statistical analysis

Statistical analyses were performed using SPSS Statistics (version 18.0), Image J and GraphPad Prism (version 8.0.2) software. Between group differences were assessed by the Mann-Whitney U-test or Student’s t-test, as appropriate. All experiments were performed more than three times, with p < 0.05 defined as statistically significant.

## Results

### Identification of oxidative stress genes in lung cancer

**Figure 2:**
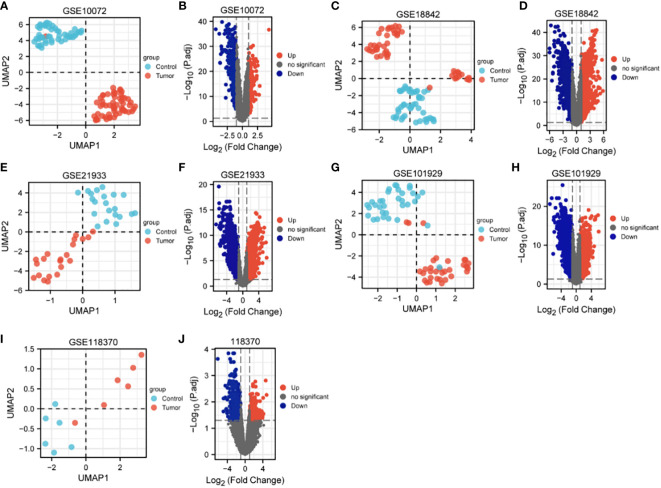
UMAP and volcano maps of the five GSE datasets (log2 (FC) |>1| and p.adj<0.05). **(A, B)** GSE10072, **(C, D)** GSE18842, **(E, F)** GSE21933, **(G, H)** GSE101929, and **(I, J)** GSE118370.

**Figure 3:**
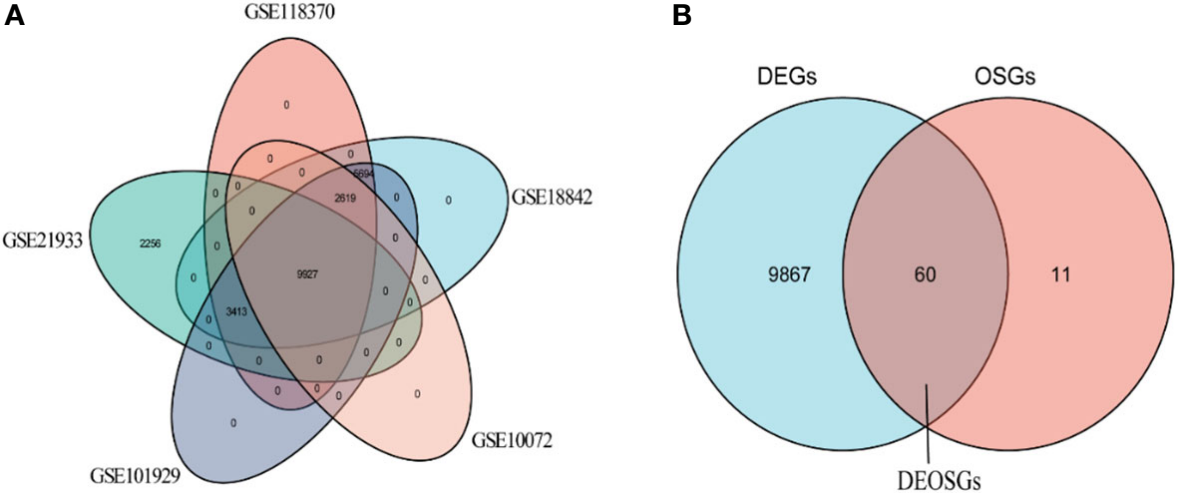
Venn diagrams for screening of **(A)** DEGs and **(B)** DEOSGs.

The characteristics of the lung cancer and normal lung tissue samples in the five GEO datasets, GSE10072, GSE18842, GSE21933, GSE101929, and GSE118370, are shown in **Table 1** and **Figure 2**. The control and tumor samples were well separated, indicating significant differentially expressed genes between the two groups. Gene expression was analyzed in tumor and normal lung tissues from the five datasets, and genes differing significantly in levels of expression were identified. Analysis of the five GSE datasets identified 9927 overlapping genes, which were defined as DEGs. In addition, 6835 OSGs downloaded from the GeneCards database were screened. Limiting the relevant score to > 10 resulted in the selection of 71 OSGs. Overlap of the 9927 DGEs and the 71 OSGs resulted in the identification of 60 differentially expressed OSGs (DEOSGs) (**Figure 3**).

**Table 1:** Characteristics of the GSE datasets.

Data Set	Platforms	Sample	Control	Tumor	General information
GSE10072	GPL96	107	49	58	Age, sex, smoking status, pathological stage
GSE18842	GPL570	91	45	46	None
GSE21933	GPL6254	42	21	21	Age, sex, pathological stage
GSE101929	GPL570	66	32	34	Age, sex, race, smoking status, smoking years, survival, survival time, pathological stage
GSE118370	GPL570	6	3	3	None

### Protein-protein interaction network and hub genes

**Figure 4:**
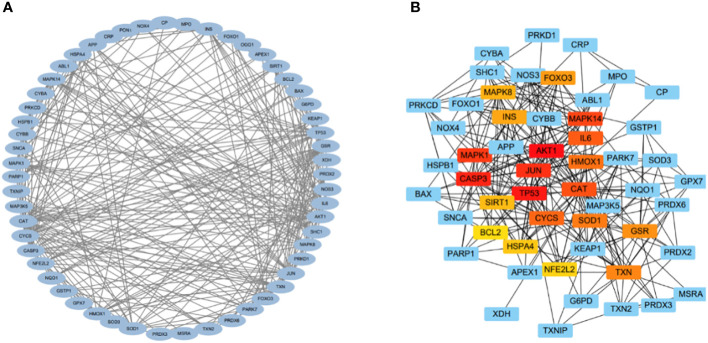
PPI and hub gene networks. **(A)** PPI network; **(B)** Hub gene network.

The relationships among DEOSGs of lung cancer were further evaluated by establishing a PPI network and screening for hub genes using Cytoscape. The top 20 genes in descending order of the MCC algorithm were identified as *AKT1, TP53, CASP3, JUN, MAPK1, MAPK14, CAT, IL6, CYCS, HMOX1, SOD1, TXN, GSR, FOXO3, INS, MAPK8, SIRT1, HSPA4, NFE2L2*, and *BCL2* (**Figure 4**; **Table 2**). The darker the color represents the more important the protein is in the interaction network.

**Table 2:** Top 20 genes, as determined by the MCC method.

Rank	Name	Score	Rank	Name	Score
1	AKT1	2648	11	SOD1	632
2	TP53	2318	12	TXN	610
3	CASP3	2130	13	GSR	594
4	JUN	2067	14	FOXO3	592
5	MAPK1	1560	15	INS	528
6	MAPK14	1374	16	MAPK8	482
7	CAT	1299	17	SIRT1	422
8	IL6	1188	18	HSPA4	402
9	CYCS	892	19	NFE2L2	366
10	HMOX1	841	20	BCL2	290

### Biological function enrichment and pathway analysis

**Figure 5:**
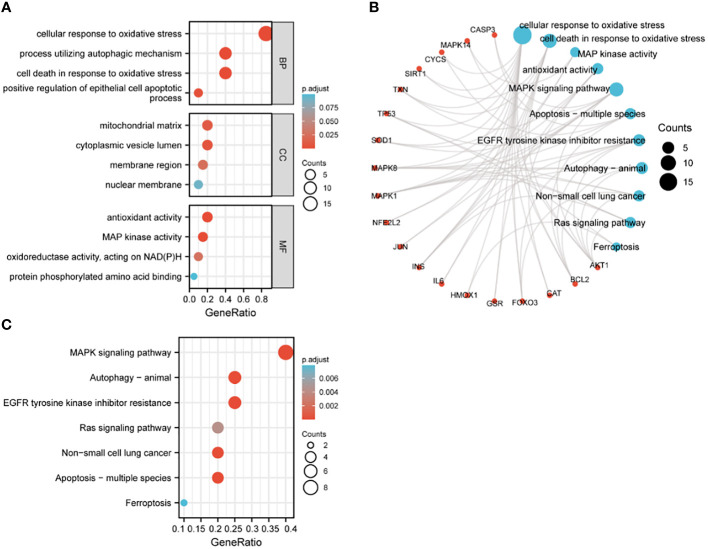
GO and KEGG enrichment analysis of hub genes. **(A)** GO analysis; **(B)** circular diagram of GO and KEGG analyses; **(C)** KEGG analysis.

**Figure 6:**
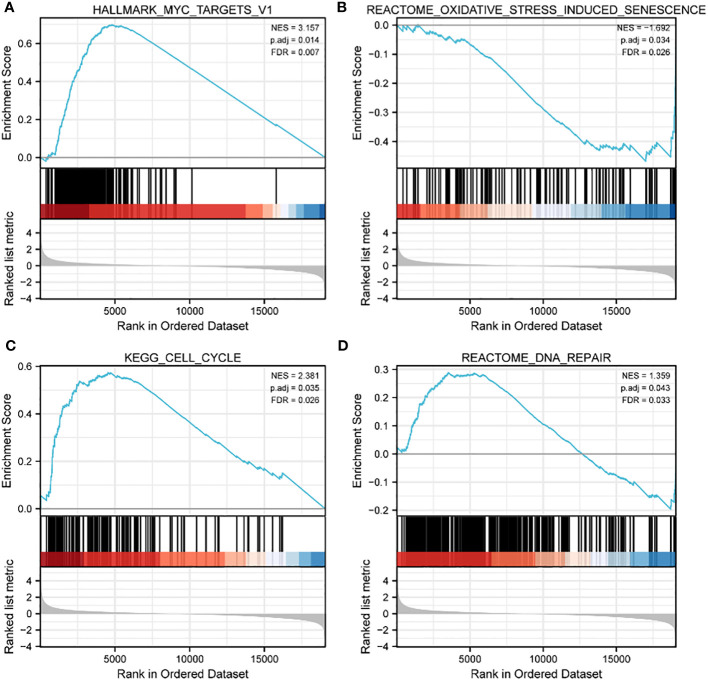
GSEA enrichment analysis of TCGA data sets. **(A)** MYC targets in HALLMARK gene sets; **(B)** oxidative stress induced senescence in C2 curated gene sets; **(C)** cell cycle in C2 curated gene sets; **(D)** DNA repair in C2 curated gene sets.

The 20 hub genes were subjected to GO and KEGG function enrichment analysis. GO analysis showed significant enrichment of biological process (BP) and molecular function terms (MF) terms. BP terms enriched significantly in lung cancers included cellular response to oxidative stress, process utilizing autophagic mechanism, cell death in response to oxidative stress and positive regulation of epithelial cell apoptotic process; whereas MF terms enriched significantly in lung cancers included antioxidant activity, MAP kinase activity, oxidoreductase activity, and protein phosphorylated amino acid binding (**Figures 5A, B**). KEGG analysis showed that significantly enriched terms included MAPK signaling pathway, autophagy-animal, EGFR tyrosine kinase inhibitor resistance, RAS signaling pathway, non-small lung cancer, apoptosis-multiple species and ferroptosis (**Figures 5B, C**). GSEA function analysis was performed to comprehensively investigate the biological functions of genes. Among the interesting items selected were MYC targets from hallmark gene sets; and oxidative stress induced senescence, cell cycle and DNA repair from C2 curated gene sets (**Figures 6A–D**).

### Univariate/multivariate Cox regression and prognostic analysis

**Figure 7:**
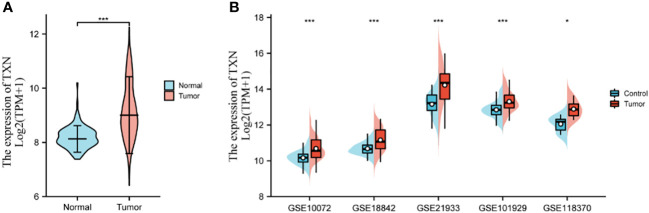
Levels of expression analysis of TXN in **(A)** the TCGA dataset and **(B)** the five GSE datasets. *p < 0.05, ***p < 0.001.

Key genes related to prognosis in patients with lung cancer were identified by analyzing differences expression of the 20 hub genes in the five GSE datasets and by KM survival analysis of the 20 hub genes in the TCGA dataset. The levels of expression of five genes, *CASP3, CAT, TXN, GSR*, and *HSPA4*, differed significantly in lung tumor and normal lung tissue samples in each GSE dataset (P < 0.05) (**Supplemental Figure 1**), whereas KM survival analysis found that the levels of four genes, *IL-6, CYCS, TXN*, and *BCL2* correlated significantly with prognosis in patients with lung cancer (**Supplemental Figure 2**). TXN expression was associated with both analyses, suggesting that TXN may be an optimal target in lung cancer treatment. The levels of expression of TXN in each dataset are shown in **Figure 7**. Obviously, the expression of TXN in the tumor group was higher than that in the control group, implicating TXN as a cancer promoting factor.

After identifying TXN as the target gene, univariate and multivariate Cox regression model were established. Univariate Cox regression analysis showed that age (P = 0.022), T stage (P < 0.001), N stage (P < 0.001), M stage (P < 0.001), pathologic stage (P < 0.001), curative outcome (P < 0.001) and TXN expression (P = 0.024) were prognostic of survival in lung cancer patients, whereas gender was not. Multivariate Cox regression analysis showed that T stage (P = 0.006, hazard ratio [HR] 1.779, 95% confidence interval [CI]: 1.178-2.286), N stage (P = 0.010, HR 2.180, 95% CI: 1.204-3.945), curative outcome (P < 0.001, HR 0.237, 95% CI: 0.168-0.336) and high TXN expression (P = 0.018, HR 1.403, 95% CI: 1.061-1.855) were independent risk factors for prognosis in patients with lung cancer (**Table 3**).

**Table 3:** Univariate/multivariate Cox regression.

Characteristics	Univariate analysis		Multivariate analysis	
	HR (95% CI)	P value	HR (95% CI)	P value
Age (≤65/>65 yrs)	1.265 (1.034-1.548)	**0.022**	1.096 (0.831-1.445)	0.517
Gender (Female/Male)	1.164 (0.949-1.428)	0.145		
T stage (T1-T2/T3-T4)	1.889 (1.480-2.412)	**<0.001**	1.779 (1.178-2.686)	**0.006**
N stage (N0-N1/N2-N3)	1.799 (1.372-2.357)	**<0.001**	2.180 (1.204-3.945)	**0.010**
M stage (M0/M1)	2.269 (1.439-3.577)	**<0.001**	1.814 (0.884-3.723)	0.105
Pathologic stage (I-II/III-IV)	2.011 (1.614-2.506)	**<0.001**	0.814 (0.451-1.469)	0.494
Curative outcome (PD/SD, PR, CR)	0.269 (0.204-0.354)	**<0.001**	0.237 (0.168-0.336)	**<0.001**
TXN (Low/High)	1.257 (1.031-1.534)	**0.024**	1.403 (1.061-1.855)	**0.018**

The bold values indicate statistically different (P < 0.05).

**Figure 8:**
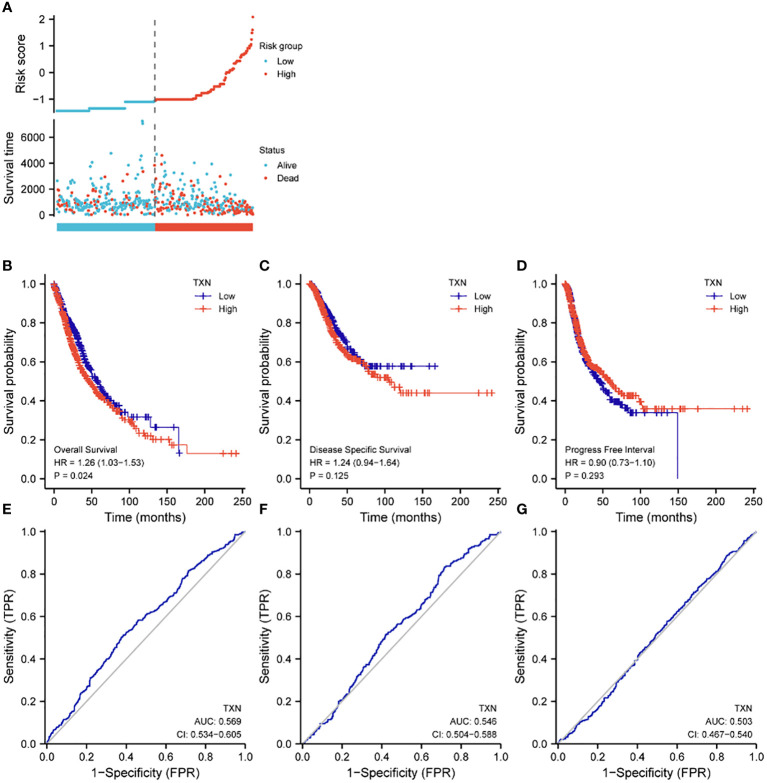
Effects of TXN expression on survival outcomes in lung cancer patients, as determined by KM survival analysis and ROC curve analysis. **(A)** Relationships of TXN expression with risk scores and individual patient survival; **(B–D)** KM analysis of the relationships between TXN expression and patient **(B)** OS, **(C)** DSS and **(D)** PFI. **(E–G)** ROC curve analysis of the relationships between TXN expression and patient **(E)** OS, **(F)** DSS and **(G)** PFI.

Assessments of the effects of TXN expression on survival of lung cancer patients showed that TXN was associated with OS (P = 0.024, HR 1.26, 95% CI: 1.03-1.56), but its predictive power was weak (AUC 0.568, 95% CI: 0.534-0.605). TXN expression was not associated with disease specific survival (DSS) or progression free interval (PFI), with both having low areas under their ROC curves (AUC) (**Figure 8**).

**Figure 9:**
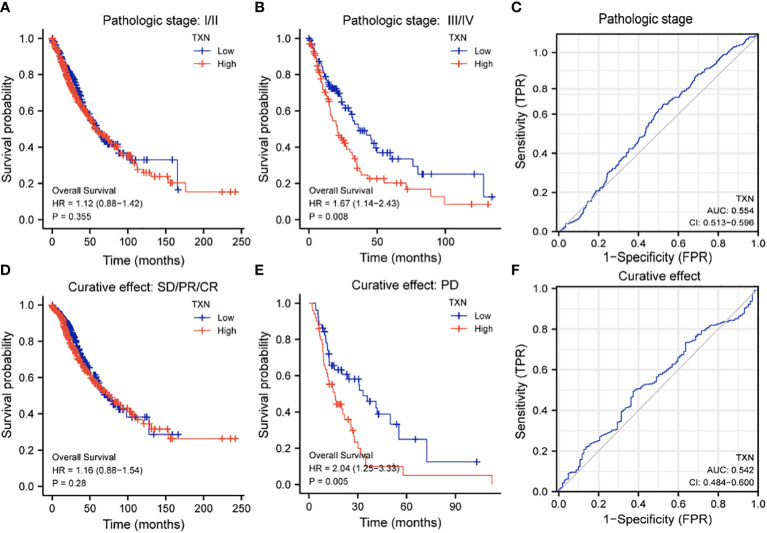
Relationships between TXN expression with T-stage and curative outcomes in lung cancer patients. **(A, B)** KM survival analysis of the relationships between TXN expression and survival in patients with T-stage **(A)** I/II and **(B)** III/IV tumors. **(C)** ROC curve analysis of the relationship between TXN expression and T stage. **(D, E)** KM survival analysis of the relationships between TXN expression and patients who achieved **(D)** SD, PR, and CR and **(E)** PD. **(F)** ROC curve analysis of the relationship between TXN expression and patient curative outcomes.

The relationships between TXN expression and pathological characteristics of patients with lung cancer were further evaluated by KM survival analysis. High expression of TXN was significantly associated with T stage (III-IV vs I-II; P = 0.008, HR 1.67, 95% CI: 1.14-2.43) and curative outcomes (progressive disease [PD] vs. stable disease [SD], partial response [PR] and complete response [CR]; P = 0.005, HR 2.04, 95% CI: 1.25-3.33) (**Figure 9**; **Supplemental Figure 3**).

### TXN expression in tissues and cells

**Figure 10:**
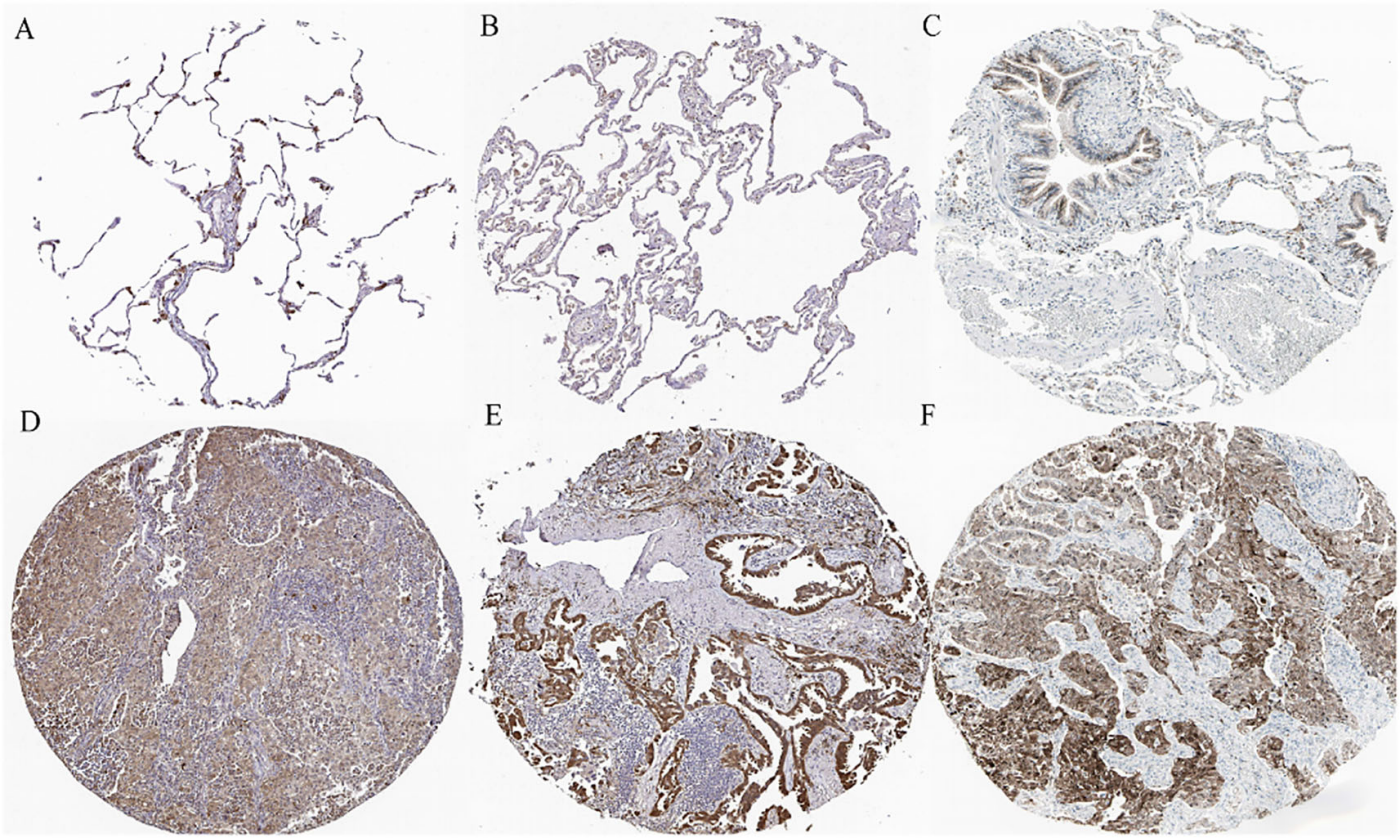
Representative immunohistochemical staining of TXN in **(A–C)** normal lung tissue and **(D–F)** lung cancer tissue samples.

**Figure 11:**
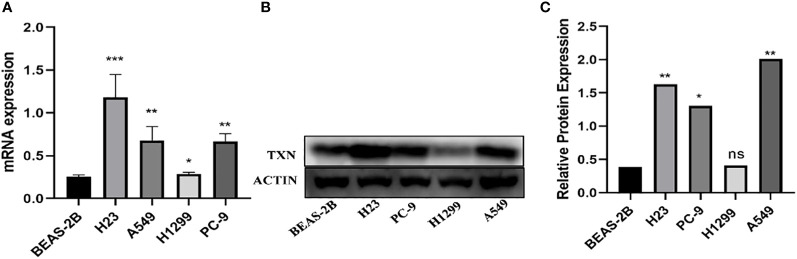
Levels of TXN mRNA and protein expression in lung cancer and normal lung epithelial cell lines. **(A)** TXN mRNA expression; **(B)** TXN protein expression; **(C)** gray value analysis of TXN protein expression. *p < 0.05, **p < 0.01,***p < 0.001, ns, no statistical difference (p > 0.05).

The immunohistochemical levels of expression of TXN in normal lung and lung cancer tissues were obtained from the HPA database. The proportion of cells positive for TXN was higher in lung cancer tissues (**Figure 10**). Evaluation of cell lines showed that TXN mRNA and protein expression levels were higher in the lung cancer cell lines than in the normal lung epithelial cell line (**Figure 11**).

### Lentiviral transfection and cell proliferation

**Figure 12:**
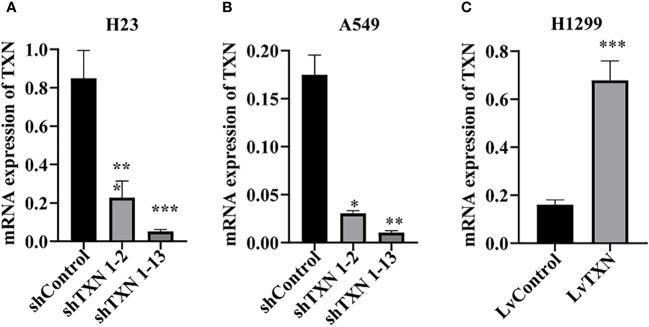
RT-qRCR verification of lentiviral TXN knockdown and overexpression. **(A)** mRAN expression of TXN after knockdown in H23; **(B)** mRAN expression of TXN after knockdown in A549; **(C)** mRAN expression of TXN after overexpression in H1299. *p < 0.05, **p < 0.01,***p < 0.001.

**Figure 13:**
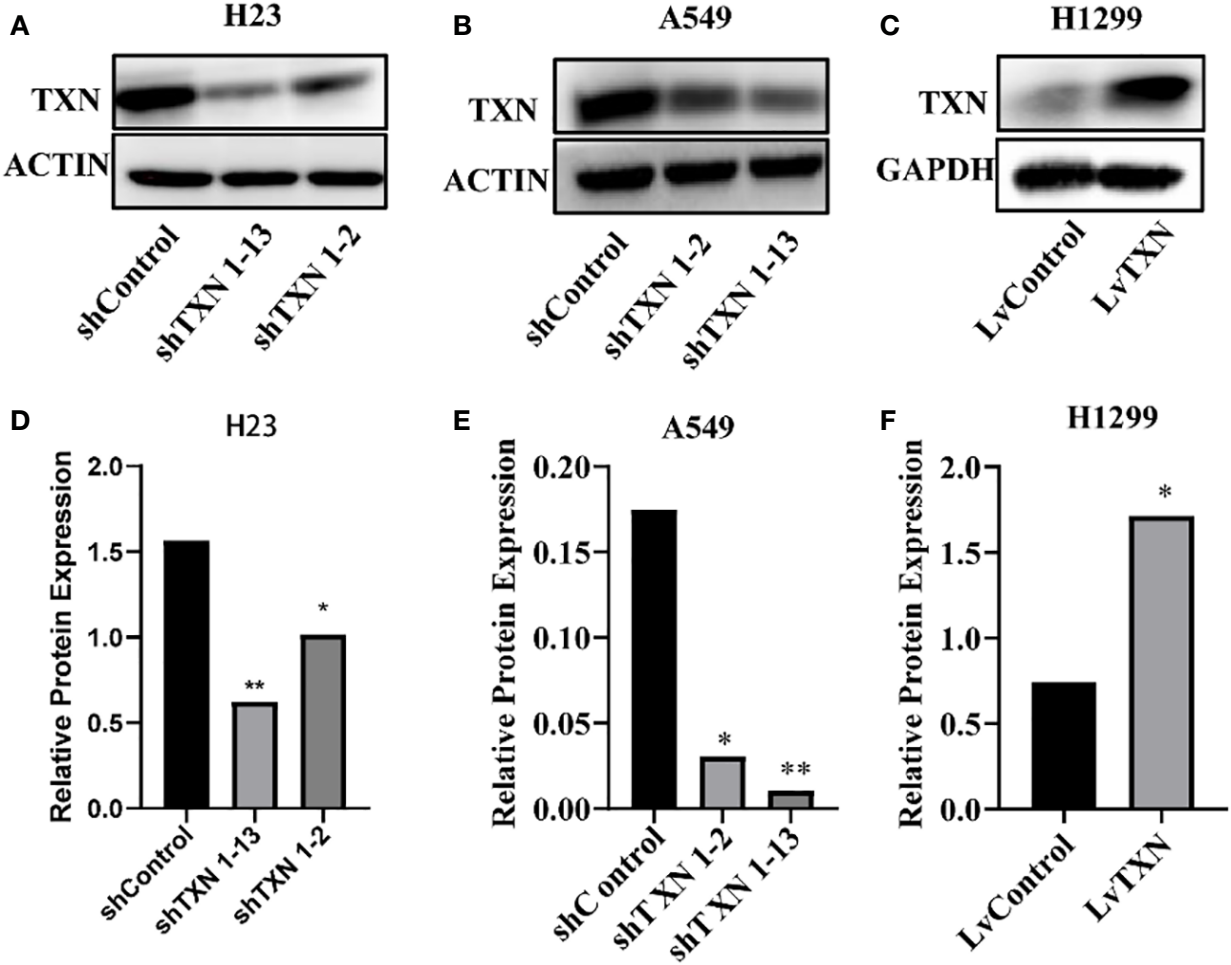
Western blot verification of lentiviral TXN knockdown and overexpression. **(A)** protein expression of TXN after knockdown in H23; **(B)** protein expression of TXN after knockdown in A549; **(C)** protein expression of TXN after overexpression in H1299. **(D–F)** Gray scale band analysis. *p <0.05, **p < 0.01.

**Figure 14:**
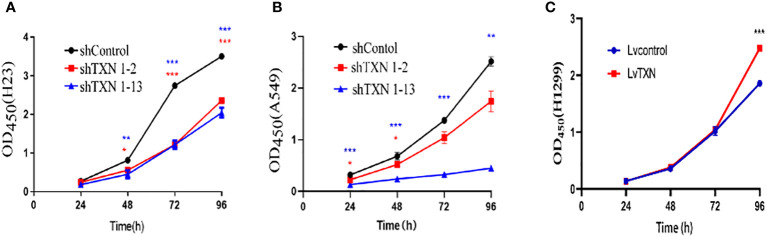
CCK-8 methods measuring the effect of TXN knockdown and overexpression on cell proliferation. **(A)** TXN knockdown in H23; **(B)** TXN knockdown in A549; **(C)** TXN overexpression in H1299. *p < 0.05, **p < 0.01,***p < 0.001.

TXN expressions were higher in H23 and A549 cells and lower in H1299 cell, therefore, knockdown assay was performed in H23 and A549 cells, and overexpression assay was performed in H1299 cell. The mRNA and protein levels were well knocked down and overexpressed (**Figures 12**, **13**). The proliferation ability was attenuated after knockdown of TXN and was enhanced after overexpression of TXN, suggesting that TXN could promote the proliferation of lung cancer (**Figure 14**).

## Discussion

Lung cancer is the leading cause of cancer deaths worldwide (2). Advances in treatment have enabled more personalized therapy, significantly improving the OS of lung cancer patients (10). The biology of lung cancer plays a prominent role in responses to treatment, especially to targeted therapy and immunotherapy. Targeted tumor-suppressing drugs based on biomarkers predicted from genetic and molecular analyses have been utilized clinically, yielding excellent results (11). Biomarkers for cancer diagnosis, management and prognosis have been identified by various methods, including high-throughput sequencing, microarray, and mass spectrometry. Publicly available databases have shown prospective potential for mining of biological information of cancers (12). In the present study, several datasets from the TCGA and GEO databases were selected, with screening of differentially expressed and hub genes identifying TXN as a target gene in lung cancer. Cox regression analysis, functional enrichment, and analyses of survival and prognosis showed that TXN was predictive of the diagnosis and prognosis of lung cancer.

TXN, a member of the thioredoxin family, is a 12 kDa molecule that acts as an antioxidant. TXN is a constituent of a redox system, which also consists of thioredoxin reductase 1 (TrxR1) and NADPH (13). Specifically, TXN contains a Cys-Gly-Pro-Cys catalytic site, which switches between thiol and disulfide conditions under the influence of TrxR1 and NADPH, thus constituting a reversible redox system (14). TXN has various biological functions, such as repairing DNA damage (15), activating transcription factors (8, 16), inhibiting apoptosis signaling (17), and eliminating reactive oxygen species (ROS) (16, 18), enabling cells to resist oxidative stress while enhancing cell proliferation and invasion. Oxidative stress increases ROS, leading to an imbalance between oxidation and antioxidation. Antioxidation is required to maintain normal cell morphology and function (19), preventing the abnormal proliferation of cancer cells (20, 21). In addition to being an important biomarker of oxidative stress, the present study utilized bioinformatics to identify TXN as a molecular target of oxidative stress in lung cancer by mining of genes related to both lung cancer and oxidative stress. Evaluation of the TCGA dataset and the five GSE datasets found that TXN expression was higher in tumor than in normal tissues, consistent with previous findings (22), indicating a correlation between high TXN expression and lung cancer.

The biological functions and clinical significance of TXN in lung cancer were further assessed by in-depth analyses. Evaluations of the correlations of TXN expression with clinicopathological characteristics and OS showed that age, TNM stage, pathological stage, curative outcomes and TXN expression correlated significantly with OS. Multivariable analysis showed that T stage (T3/T4), N stage (N2/N3), pathological stage (III/IV), curative outcomes (PD), and high expression of TXN were key risk factors for lung cancer survival. A previous bioinformatics study identifying six prognostic biomarkers in lung cancer bioinformatics found, by Cox multivariate analysis that pathological stage (II/III/IV) was independently prognostic of OS and DFS (23). TNM stage has shown to be prognostic in various cancers, including lung (24, 25), liver (26), rectal (27), pancreatic (28), and skin (29) cancers. TNM stage is also associated with the risk of tumor recurrence and with treatment methods (30). In addition, the efficacy of treatment among patients with the same type of cancer has been found to be dependent on individual differences. A deep learning model that included PD, distant metastasis and local recurrence showed good performance in predicting prognosis of lung cancer patients (31).

In addition to its regulation of redox balance, TXN can interact with different proteins to regulate tumor development through redox-dependent signaling pathways (8). The present study found that biological functions identified during enrichment analysis included oxidative stress, DNA damage repair, cell death, cell cycle, and protein phosphorylation. TXN was found to act through the MAPK and RAS signaling pathways and to target MYC. Defects in DNA damage repair signaling contribute to the occurrence of tumors (32). TXN can sustain the activity of ribonucleic acid reductase (RNR), a rate-limiting enzyme during DNA replication and repair, enhancing DNA synthesis. Tumor survival requires the inhibition of the death of intact cancer cells and promoting the death of damaged cancer cells, with TXN playing this role in cancer cell apoptosis, autophagy, and ferroptosis. β-Lapachone was reported to induce the apoptosis of HL-60 cells by inhibiting TrxR and further increasing oxidative stress (33), and TrxR inhibition was found to increase the level of oxidative stress due to a reduction in the relative amount of TXN (34). TXN was shown to inhibit ferroptosis during adriamycin induced cardiotoxicity through mTORC1 signal activation (35). Impaired cell cycle in cancer leads to the proliferation of malignant cells (36). Exogenous Trx was found to inhibit G2/M cell cycle arrest and apoptosis in renal tubular cells (37). The RAS/MAPK signaling pathway plays an important role in tumor development through protein phosphorylation cascade reactions (38). Trx was also shown to prevent hyperoxia-induced lung injury caused by the loss of UCP2 through the mkk4-p38mAPK-pgC1α signaling pathway (39). In addition, the MAPK (ERK1/2 and ERK5) pathway was found to stabilize MYC protein (40). GSEA analysis in the present study showed enrichment of the MYC target, suggesting that TXN may target the MYC protein through the MAPK signaling pathway, promoting the occurrence and development of lung cancer. Additional studies, however, are needed to confirm these findings.

Prognostic analysis in the present study showed that high TXN expression was associated with poor prognosis in patients with lung cancer. Its AUC, however, was only 0.567, suggesting that TXN is weakly predictive of patient prognosis. Lung cancer prognosis, however, has been associated with many clinical and genetic factors (41). Single genes are weakly predictive of the prognosis of patients with lung cancer, with evaluations of single genes having limited clinical benefits. Although targeting the mutant form of EGFR has shown significant benefits (42), long-term treatment with targeted agents can result in drug resistance, relapse, and metastasis. Multifactorial models, rather than single genes or single pathological characteristics, are required to identify highly sensitive and accurate reliable prognostic markers in lung cancer.

This study had several limitations. First, it did not comprehensively screen sufficient numbers of lung cancer related datasets. The five GSE datasets in our study were determined after previewing the topic, population, clinical characteristics, grouping, intervention and other items. Therefore, many data sets may have been omitted owing to subjective factors. Second, because these datasets differed in sample size and clinical characteristics, some factors related to patient prognosis may have been omitted, weakening the predictive power of the included factors. Finally, all the genes included in this analysis were protein encoding genes. Thus, the predictive information of some non-protein coding genes may have been missed. Despite these limitations, however, this study may have clinical significance. Evaluation in large numbers of patients from multiple centers may identify additional potential targets for the diagnosis, management and prognosis of lung cancer.

In conclusion, the present study, which utilized bioinformatics methods to identify lung cancer oxidative stress related genes, found that TXN was related to the prognosis of lung cancer and had certain predictive value. TXN was also found to be more highly expressed in lung cancer than in normal lung epithelial cell lines. TXN may be a tumor-suppressing therapeutic target in lung cancer.

## Data availability statement

The datasets presented in this study can be found in online repositories. The names of the repository/repositories and accession number(s) can be found in the article/**Supplementary Material**.

## Author contributions

XD conceived and designed the study. XL performed the research and wrote the paper. YH polished the language of the manuscript. YF performed data analysis. All authors participated in the review of the manuscript content and the revision of the format. All authors contributed to the article and approved the submitted version.
