# Supplementary figures and images for: Identification of thioredoxin-1 as a biomarker of lung cancer and evaluation of its prognostic value based on bioinformatics analysis
Source: Front Oncol. 2023 Jan 27;13:1080237. doi: 10.3389/fonc.2023.1080237 (PMC9911911; doi:10.3389/fonc.2023.1080237)

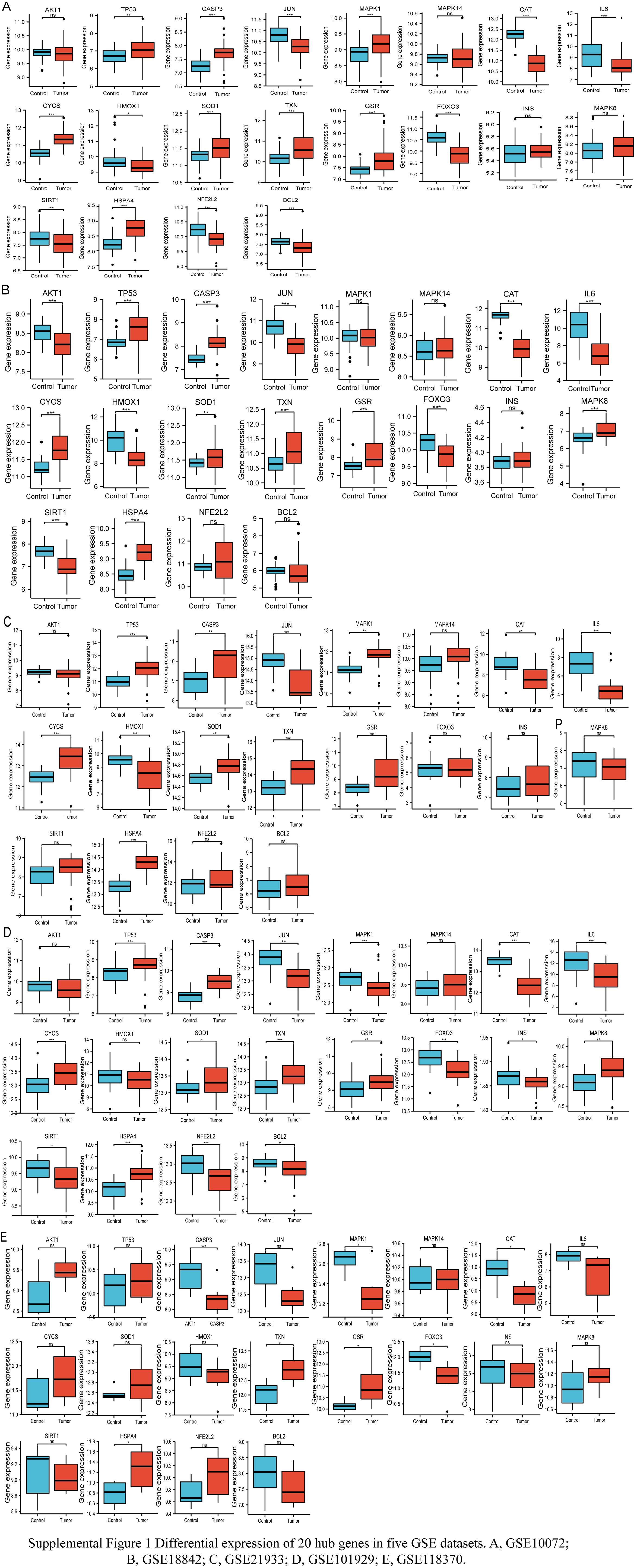

Supplement: Supplementary file 1 [file DataSheet_1.zip › Supplemental Figure 1.tif]

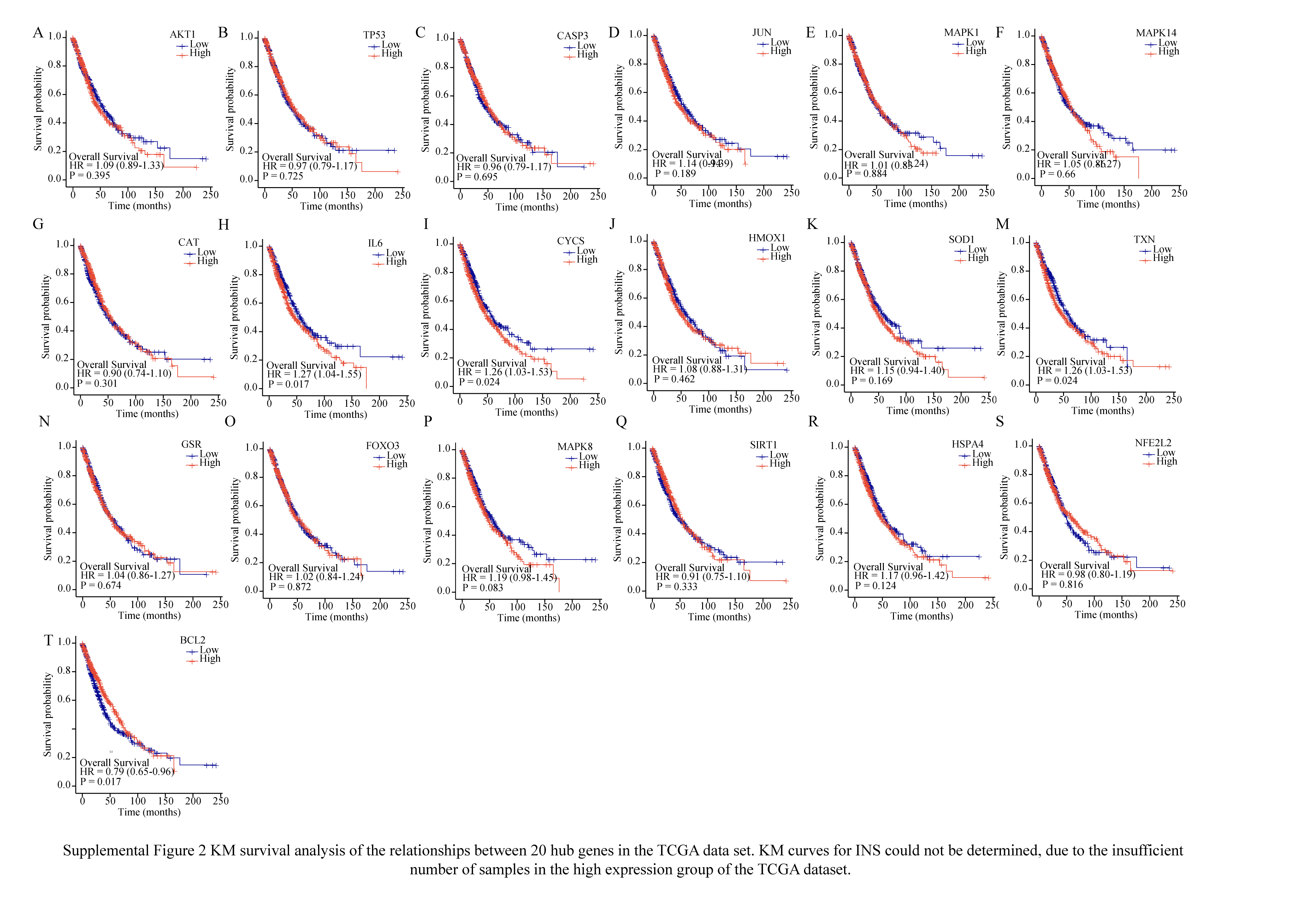

Supplement: Supplementary file 1 [file DataSheet_1.zip › Supplemental Figure 2.tif]

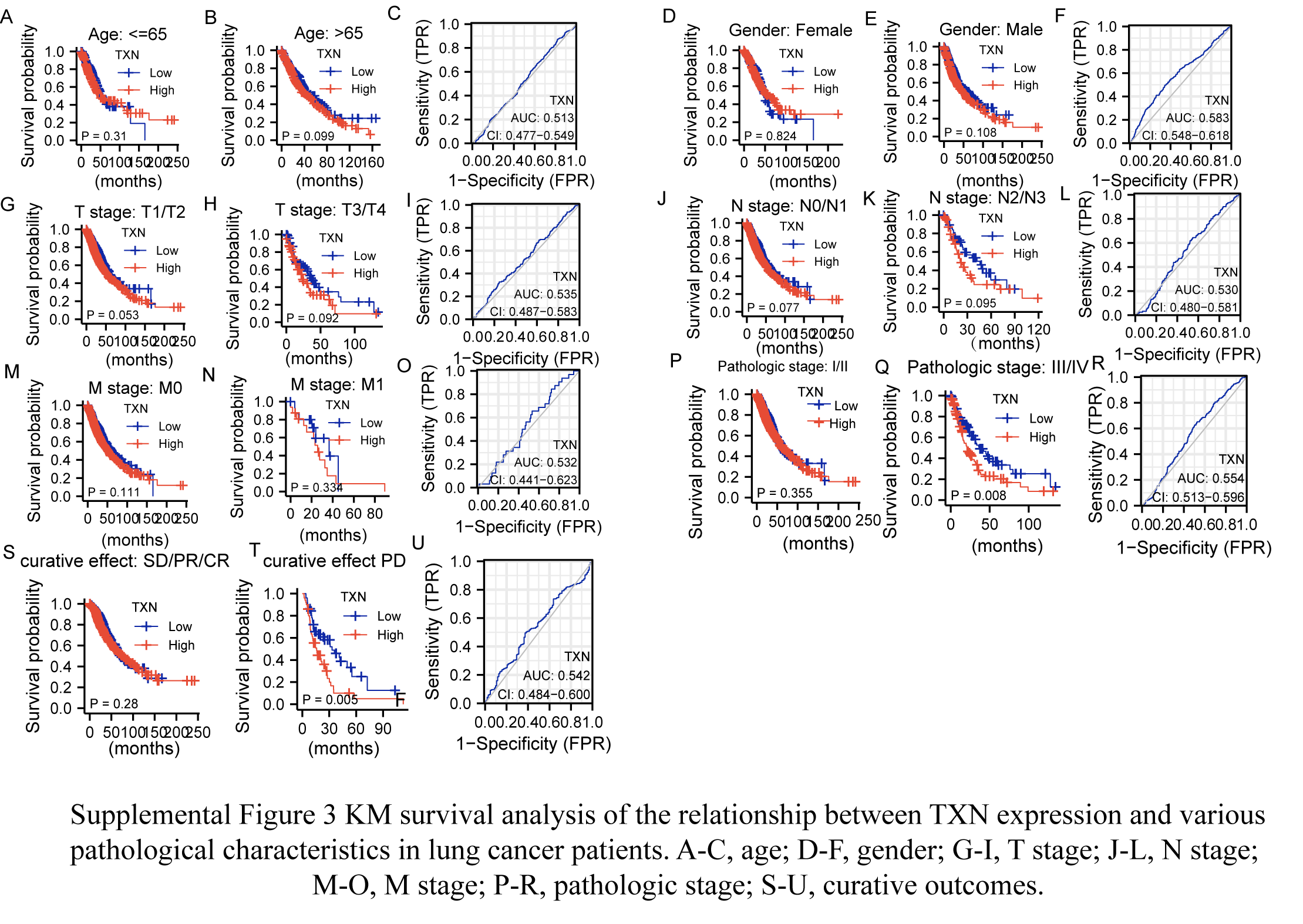

Supplement: Supplementary file 1 [file DataSheet_1.zip › Supplemental figure3.tif]
